# Supplementary material for: Cumulative Weighing of Time in Intertemporal Tradeoffs
Source: J Exp Psychol Gen. 2016 Sep;145(9):1177–205. doi: 10.1037/xge0000198 (PMC4998108; doi:10.1037/xge0000198)
Supplement: Supplementary file 1 [file Scholten_Individuals.zip › plots/e29_p401_eg2_priors_and_posteriors.pdf]

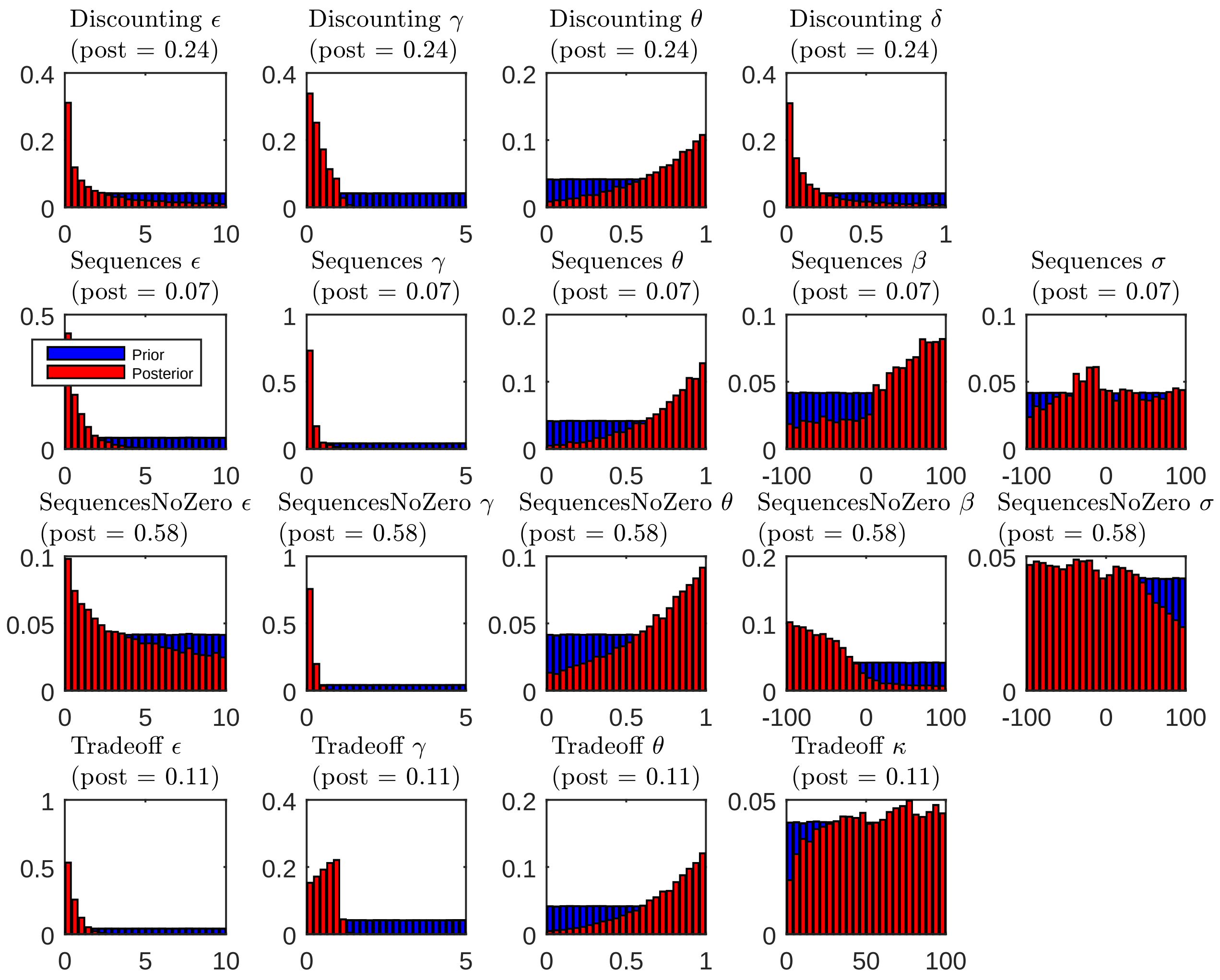
Discounting ϵ
(post = 0.24)
Discounting γ
(post = 0.24)
Discounting θ
(post = 0.24)
Discounting δ
(post = 0.24)
Sequences ϵ
(post = 0.07)
Prior
Posterior
Sequences γ
(post = 0.07)
Sequences θ
(post = 0.07)
Sequences β
(post = 0.07)
Sequences σ
(post = 0.07)
SequencesNoZero ϵ
(post = 0.58)
SequencesNoZero γ
(post = 0.58)
SequencesNoZero θ
(post = 0.58)
SequencesNoZero β
(post = 0.58)
SequencesNoZero σ
(post = 0.58)
Tradeoff ϵ
(post = 0.11)
Tradeoff γ
(post = 0.11)
Tradeoff θ
(post = 0.11)
Tradeoff κ
(post = 0.11)
